# Fertility and Markers of Male Reproductive Function in Inuit and European Populations Spanning Large Contrasts in Blood Levels of Persistent Organochlorines

**DOI:** 10.1289/ehp.10700

**Published:** 2007-11-23

**Authors:** Jens Peter Bonde, Gunnar Toft, Lars Rylander, Anna Rignell-Hydbom, Aleksander Giwercman, Marcello Spano, Gian Carlo Manicardi, Davide Bizzaro, Jan K. Ludwicki, Valentina Zvyezday, Eva C. Bonefeld-Jørgensen, Henning Sloth Pedersen, Bo A.G. Jönsson, Ane Marie Thulstrup, INUENDO

**Affiliations:** 1 Department of Occupational Medicine, Åarhus University Hospital, Århus, Denmark; 2 Division of Occupational and Environmental Medicine and Psychiatric Epidemiology, Lund University Hospital, Lund, Sweden; 3 Reproductive Medicine Center, Lund University, Malmö University Hospital, Malmö, Sweden; 4 Section of Toxicology and Biomedical Sciences, BAS-BIOTEC-MED, ENEA Casaccia, Rome, Italy; 5 University of Modena and Reggio Emilia, Modena, Italy; 6 Institute of Biology and Genetics, Politechnical University of Marche, Ancona, Italy; 7 Department of Environmental Toxicology, National Institute of Hygiene, Warsaw, Poland; 8 Kharkiv State Medical University, Kharkiv, Ukraine; 9 Institute of Public Health, Department of Occupational and Environmental Medicine, University of Aarhus, Århus, Denmark; 10 Centre for Arctic Environmental Medicine, Nuuk, Greenland; 11 http://www.inuendo.dk

**Keywords:** Inuit, polymorphisms, reproductive health, semen quality, sex hormone receptors, time to pregnancy, xenobiotics

## Abstract

**Objective:**

We synthesized the main findings from an international epidemiologic study on the impact of biopersistent organic pollutants (POPs) on human reproductive function.

**Data sources and extraction:**

We used a database with interview and biological data from 2,269 women and their spouses, and 18 published core papers.

**Data synthesis:**

The study did not provide direct evidence of hormone-like activity of the polychlorinated biphenyl (PCB) congener CB-153 and the main dichlorodiphenyltrichloroethane (DDT) metabolite, 1,1-dichloro-2,2-bis(*p*-chlorophenyl)ethylene (*p,p*′-DDE), as serum concentrations of these compounds were not consistently related to either endogenous or exogenous hormone activity in serum. Nevertheless several links bewteen POP exposure and biomarkers of male reproductive function were identified. First, an association between high CB-153 serum levels and low sperm counts was detected within a subgroup of men with short androgen receptor CAG repeat length. Second, a relationship between increased CB-153 serum concentrations and decreased sperm motility was seen in all four studied regions, and indications of reduced neutral α-glucosidase activity in seminal plasma point to a post-testicular effect. Third, damage of sperm chromatin integrity was considerably less frequent in Greenlandic Inuits compared with that in European groups, and only in the latter was impairment of sperm chromatin integrity related to POPs. Despite these effects, fertility in terms of time taken to conceive was not related to POPs except in Inuits. A likely explanation of the latter was not identified.

**Conclusions:**

POPs may interfere with male reproductive function without major impact on fertility. The data do not provide direct evidence for endocrine disruption, hence other mechanisms should also be considered.

Subfertility is encountered among some 15% of all couples trying to conceive (Juul et al. 1999), and sperm counts in the subfertile range are prevalent in some countries (Carlsen et al. 2005). Although advances in understanding of causes and mechanisms have been made during the past few decades (Aitken and Baker 2004; DeMasters 2004; Sharpe 2003), major gaps in knowledge still preclude effective prevention of the majority of infertile cases. Lessons learned from the occupational arena and indications of contemporary declining trends and geographic variation in the occurrence of male reproductive disorders have fueled the search for environment- and lifestyle-related risk factors. The hypothesis that ubiquitous man-made chemicals in the environment may interfere with proper development of reproductive organs through interaction with natural hormonal regulation has achieved much attention (Toppari 2002). Proponents of the hypothesis focus on *a*) an apparent decline of male reproductive health and on evidence that several male reproductive disorders are of fetal origin and may share common causes (Skakkebaek et al. 2001); *b*) that similar developmental disorders of male reproductive organs can be induced in experimental studies after exposure to various compounds that interfere with hormonal homeostasis (Toppari 2002); and *c*) that reproductive anomalies have been documented among sons of mothers treated during pregnancy by the potent synthetic estrogen diethylstilbestrol (Sharpe and Skakkebæk 1993). Opponents argue that the evidence on major temporal shifts of, for example, sperm counts, is circumstantial (Handelsman 2001) and that the large number of xenobiotics detectable in human tissues have low hormonal potencies (Daston et al. 1997) and occur in concentrations far below levels that conceivably could have a major impact on reproductive organ development (Safe 2004). Updated comprehensive reviews conclude that current experimental and epidemiologic evidence does not with sufficient certainty support the view that environmental endocrine disruptors contribute to an increase in male reproductive disorders; neither does it provide sufficient grounds to reject this hypothesis (Storgaard et al. 2005; Vidaeff and Sever 2005). More recently, attention has shifted from direct xenobiotic agonistic or antagonistic actions on the human estrogen or androgen receptor to interference with endogenous hormonal regulation (Sharpe and Irvine 2004).

Epidemiologic studies explicitly designed to corroborate or refute the environmental hormone hypothesis are few, but such studies are important in providing reliable answers as to whether xenobiotics are among important preventable causes of subfertility and other reproductive disorders. From 2002 to 2005 a European Union (EU) Fifth Framework Programme Research and Development project, INUENDO, was carried out explicitly to address this deficit in current knowledge. The main findings have been published in a number of focused original papers (Axmon et al. 2006; Bonefeld-Jorgensen et al. 2006; Elzanaty et al. 2006; Giwercman et al. 2007; Giwercman AH et al. 2006; Giwercman et al. 2006; Jönsson et al. 2005; Kruger et al. 2007; Long et al. 2006, 2007; Spano et al. 2005; Stronati et al. 2006; Tiido et al. 2006; Toft et al. 2004, 2005a, 2005b, 2006b, 2007). The purpose of this article is to provide an overview of the main results that emerged from the INUENDO project and to review the findings relative to the overall evidence on persistent organic pollutants (POPs) and human fertility.

## Methods

The INUENDO studies combine four interview studies of time to pregnancy (TTP) with four cross-sectional studies of male reproductive hormones and semen quality (Table 1). Three study populations included pregnant women and their male spouses who had antenatal care visits from May 2002 through February 2004 at one of three locations: *a*) the local hospitals in 19 cities and settlements throughout Greenland, *b*) a large central hospital in Warsaw, Poland, and *c*) three hospitals and eight antenatal clinics in Kharkiv, Ukraine. A fourth study population included Swedish fishermen and fishermen’s wives. They were enrolled independent of current pregnancy in two separate steps from an existing cohort (Rignell-Hydbom et al. 2004; Rylander et al. 1995). A total of 2,269 women from the four localities were enrolled (participation rates 26–90%; Table 1). In Greenland, Kharkiv, and Sweden, the age distribution and the number of children did not differ between participants, those who declined participation, and nonrespondents. Available data did not allow for nonresponse analysis in the Polish sample (Toft et al. 2005a). Male spouses were consecutively encouraged to participate in a semen study until approximately 200 men at each site had agreed (participation rates 7–79%; Table 1). The low particpation rate among Swedish fishermen (7%) is due to the more elderly study population and the recruitment procedure that involved postal correspondence in several consecutive steps.

Information on TTP for the current (the three pregnancy–based cohorts) or the latest planned pregnancy (the Swedish population–based cohort) was obtained by in-person interviews with the women at the hospital or residence or by telephone (Sweden) using a structured questionnaire (Toft et al. 2005a). The male partners provided separate interview data on lifestyle factors, urogenital disorders, and infertility that were used to describe reproductive characteristics of couples, with men providing or not providing semen samples (Toft et al. 2005a). Finally, women and men had a venous blood sample drawn (Table 1).

Polychlorinated biphenyls (PCBs) and the dichlorodiphenyltrichloroethane (DDT) metabolite 1,1-dichloro-2,2-bis(*p-*chlorophenyl)-ethylene (*p,p*′-DDE) were selected as biomarkers of exposures of interest because of documented hormonal actions (Bonefeld-Jorgensen et al. 2001; Kelce et al. 1997; Moore et al. 1997), widespread occurrence worldwide (Bignert et al. 1998), and existence of reliable and relatively inexpensive assays suitable for large-scale epidemiologic studies. 2,2′,4,4′,5,5′-Hexachlorobiphenyl (CB-153) was selected as a marker of PCB congeners because it correlated well with both total PCB concentration in plasma and serum from Swedish subjects and Inuits from Greenland (Glynn et al. 2000; Grimvall et al. 1997) and with the 2,3,7,8-tetrachlorodibenzo-*p*-dioxin (TCDD) equivalent (TEQ) in plasma from PCB as well as the total POP-derived TEQ in plasma in American Vietnam veterans (Gladen et al. 1999). The antiandrogenic compound *p,p*′-DDE, the major metabolite of the insecticide DDT was selected as a supplementary marker of POP exposure. Moreover, xeno-hormone activity in serum cleared for endogenous hormones and dioxin-like activity in the lipid fraction of serum was measured in a subset of the study population by *ex vivo* chemical-activated luciferase gene expression (CALUX) assays. Reporter gene constructs and cell culture systems were used to explore whether integrated measures of xenobiotic receptor binding are related to reproductive end points. The luciferase activity was expressed as relative light units (RLUs) per milliter serum; reference levels were calculated to 3.31 RLU/mL serum [estrogen receptor (ER), androgen receptor (AR)] and 6.67 RLU/mL serum [aryl hydrocarbon receptor (AhR)]. For details, the reader is referred to original papers (Bonefeld-Jorgensen et al. 2006; Kruger et al. 2007; Long et al. 2006). An overview of biological markers of male reproductive function investigated in the present study is given in Table 1.

The fine tuning of the *AR* function is regulated by two polymorphic sequences in the transactivating part of the receptor, namely, polyglutamine-encoding CAG repeats and polyglycine-encoding GGN triplets (Gao et al. 1996; Tut et al. 1997). Earlier studies have indicated that the number of CAG repeats and the GGN triplets can influence the functional status of the *AR*. Analyses of interactive effects of *AR* polymorphisms were performed to provide clues as to causal inference and insight into possible mechanisms (Giwercman et al. 2007). For these analyses CAG repeat numbers were categorized into five groups of almost equal size: < 20, 20/21, 22/23, 24, and > 24.

### Data analysis

To create an overview of the cross-sectional relations between POP exposure and all reproductive end points and to enable a systematic and coherent assessment of strength, exposure response, and internal consistency of associations, we summarized the data using a simplified, uniform approach building on comprehensive statistical analyses in previous papers (Table 1).

#### Common risk estimates

We present data at the aggregated level across all regions unless inappropriate because of heterogeneous associations. Heterogeneity was examined by interaction terms in multiple regression models. However, because the frequency of gene polymorphisms regulating male reproduction seems to differ substantially between Inuits and Europeans (Giwercman et al. 2007), all analyses were stratified accordingly. With this approach, effect modification by European regions may be hidden, but region-specific associations are presented comprehensively in the core articles.

#### Exposure categorization

Central measures of distributions in terms of medians and corresponding adjusted geometric mean values are presented according to exposure levels divided into three intervals. Cutoff values were selected as trade offs between numbers in each category, contrast of exposure, and ranges within each interval for CB-153 (ng/g lipid): 0–100, 101–200, and > 200 (highest value 5,460); and for *p,p*′-DDE (ng/g lipid): 0–500, 501–1,000, and > 1,000 (highest value 13,200).

Xenobiotic CALUX values were dichotomized into values above and below the average reference value of 3.13 RLU/mL serum for ER and AR assays and 6.67 RLU/mL serum for the AhR assay. For the agonistic assays, higher CALUX values indicate agonistic transactivation, and for the competitive assays, higher values indicate enhanced (synergistically or additively) transactivation of the natural or synthetic ligands and lower values indicate an antagonistic effect on ligand-induced receptor transactivation (Bonefeld-Jorgensen et al. 2006; Kruger et al. 2007; Long et al. 2006).

#### Statistical methods

The distribution of hormonal and semen characteristics in each exposure category was compared with the reference category by multiple linear regression. In addition, tests for linear trends across the entire exposure range were performed by similar methods but with exposure entered as a continuous variable. With few exceptions, end points were transformed by the natural logarithm to normalize skewed distributions as described in the core articles. In linear trend analyses, the exposure variables were also transformed by the natural logarithm to account for the higher variability in the high end of the distribution. Adjustments for potential confounding variables included only a few well-established determinants, which were included regardless of effects in the present data set. These determinants are listed in Table 2 (footnotes). Comprehensive confounder analysis according to the change in estimate method is provided in the core articles. The parsimonious approach we use here and the comprehensive approach applied in the core articles resulted essentially in similar findings and, if not, deviances are explicitly addressed. Geometric mean values and their confidence intervals (CIs) were obtained by back transformation, whereas linear regression coefficients in analyses based on continuous exposure variables were not. All summary analyses presented in this article were performed using SAS 9.13 software (SAS Institute Inc, Cary, NC, USA).

#### Systematic criteria used to help distinguish spurious from causal associations

*A priori* hypotheses declared before the execution of the project were held in general terms. Therefore, most of the several hundred comparisons that have been performed should be considered explorative, and the risk of spurious associations regardless of statistical significance testing may be high. Evaluation of consistency of findings across regions including assessment of strength (magnitude) of associations and exposure–response relationships has been performed throughout.

## Results

### Exposure levels

**Figure 1:**
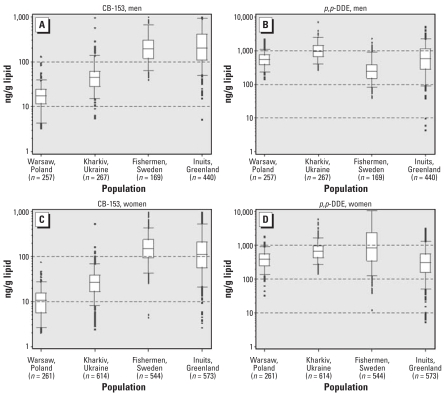
Box plots showing lipid-adjusted serum concentrations of CB-153 and *p,p*′-DDE (ng/g serum lipid) in men (*A, B*) and women (*C, D*) in four regions. Values shown are median (line within box), 25th and 75th percentiles (bottom and top of box, respectively), 5th and 95th percentiles (lower and upper bars on whisker, respectively), and outliers (circles). Numbers below region labels indicate numbers of men or women who provided blood samples.

The blood measurements of POP markers in men and women demonstrated large variations between and within study populations (Figure 1) (Jönsson et al. 2005). The median serum concentrations of the most abundant PCB congener, CB-153 as well as the DDT metabolite *p,p*′-DDE varied > 10-fold between regions. The within-region ranges of the 5th and 95th percentiles were of similar magnitude.

**Figure 2:**
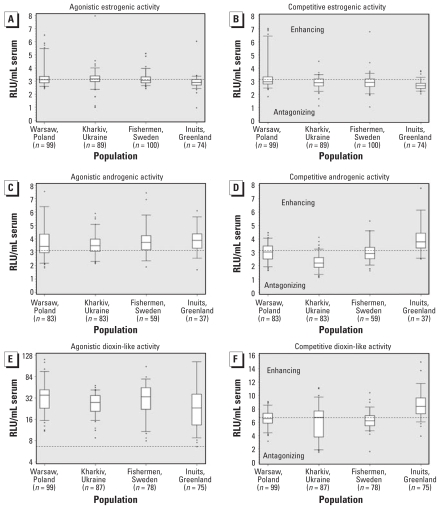
Box plots showing estrogenic (*A*, *B*), androgenic (*C, D*), and aryl hydrocarbon (dioxin-like, AhR; *E, F*) CALUX activities among men in four regions. For each receptor, agonistic assays are displayed on left panels (*A*, *C, E*) and competitive assays are displayed on right panels (*B, D, F*). Values shown are median (line within box), 25th and 75th percentiles (bottom and top of box, respectively), 5th and 95th percentiles (lower and upper bars on whisker, respectively), and outliers (circles). The dotted horizontal lines indicate the reference line. For agonistic assays, the reference line is solvent contol activity. For competitive assays, the reference line indicates competitive activity upon co-exposure to the natural (17β-estradiol) or synthetic ligands (the AR agonist R1881 and the AhR agonist TCDD) at a concentration of 40–50% of the concentration eliciting maximal response according to the assay-specific calibration curve (EC_40–50_). For competitive assays, values above the reference line indicate enhancement of ligand activity and values below the line indicate competitive inhibition. Numbers below region labels indicate number of male measurements.

The INUENDO project is the first large-scale population-based epidemiologic study that has evaluated the integrated xenohormone (ER, AR) and dioxin-like (AhR) activity in serum by CALUX assays (Bonefeld-Jorgensen et al. 2006; Kruger et al. 2007; Long et al. 2006). These assays demonstrated agonistic as well as competitive receptor interference of serum cleared for endogenous hormones, although the between- and within-region variation was small compared with variations in POP concentrations (Figure 2). The CALUX activities were only weakly correlated with CB-153 and *p,p*′-DDE, indicating that these organochlorines are not the important contributors to the measured xenobiotic serum activity.

### Reproductive end points according to polychlorinated biphenyls (CB-153) and *p,p*′-DDE

Fecundability measured by TTP in couples that conceived was not related to CB-153 among Europeans, but among Inuits the fecundability was reduced among intermediate- and high-level exposed men and women compared with those exposed to low levels of CB-153, although no obvious exposure–response relations were found and findings were of borderline significance (Axmon et al. 2006). The risk associated with male exposure was most likely not confounded by female exposure and vice versa, but because of the strong correlation between CB-153 and *p,p*′-DDE among Inuits, it was not possible to determine if the risk was associated with CB-153 or *p,p*′-DDE or an interaction between the two compounds.

None of the five male reproductive hormones measured in serum varied consistently with CB-153 serum levels among Inuits and Europeans, but luteinizing hormones increased with increasing CB-153 among Inuits and free testosterone decreased, whereas sex hormone–binding globulin levels became higher with increasing CB-153 among European men (Table 2). Comprehensive additional analyses of reproductive hormones within each region indicated several endocrine responses associated with CB-153 blood levels in some regions but not in others (Giwercman AH et al. 2006).

Sperm count and the proportion of morphologically normal sperm was not related to CB-153 in any study group, but progressive sperm motility was inversely related to CB-153 in serum among both Inuits and Europeans, with consistent indications of exposure response relationships (Table 2; Toft et al. 2006). The percentage of progressively motile sperm decreased by 3.6% (95% CI, 1.7–5.6) per 1 U increase in the logarithm of serum CB-153 (nanograms per gram lipid) in the entire data set (Toft et al. 2006).

We observed significant interactive effects of CB-153 and *AR* CAG repeats on sperm counts but not on semen volume, sperm motility, or sperm morphology (Giwercman et al. 2007). Thus, when total sperm number was compared in men with CB-153 levels above median and those with exposure below median, sperm output was 40% lower in subjects with *AR* CAG repeat length < 20 but not in those with ≥ 20 repeats.

Two independent indicators of sperm chromatin integrity [% DNA fragmentation index (%DFI) and terminal deoxynucleotidyl transferase dUTP nick end-labeling (TUNEL)] were strongly related to CB-153 serum levels among Europeans but not among Inuits (Table 2; Spano et al. 2005; Stronati et al. 2006). Among Europeans the proportion of sperm with impaired sperm chromatin integrity increased with increasing CB-153 blood concentrations to a level almost double that in the high-level-exposed group. This association seemed not to be confounded by concomitant exposure to *p,p*′-DDE. Our study failed to demonstrate any relation between CB-153 and apoptotic sperm biomarkers (Fas and Bcl-xL) in any region or overall (Stronati et al. 2006). No interactive effects of *AR* polymorphisms and CB-153 on %DFI were found (Giwercman et al. 2007).

Associations between CB-153 and the proportion of Y spermatozoa exhibited strong heterogeneity across regions (Tiido et al. 2006). In the Swedish fishermen cohort, CB-153 serum levels were positively associated with the proportion of Y chromosome–bearing spermatozoa [linear regression coefficient β = 0.53 (95% CI, 0.001 to 1.05)], whereas among Polish men, levels of CB-153 correlated negatively with the proportion of Y sperm [β = −0.54 (95% CI, −0.92 to −0.14)]. The difference in average proportion Y-bearing sperm between these groups was small (51.2% for the fishermen sample, 50.3% for the Polish sample).

None of several seminal markers of epididymal and accessory sexual gland function varied consistently with CB-153 serum levels among Inuits or Europeans across all regions (Table 2; Elzanaty et al. 2006). However, a statistically significant negative association between the levels of CB-153 and the total activity of NAG was seen among men from Greenland and Warsaw and in the entire data set (Elzanaty et al. 2006). Moreover, NAG was also lower in high-level-exposed Swedish fishermen, whereas in Kharkiv, higher levels of CB-153 tended to be related to higher levels of NAG. Associations between serum concentrations of *p,p*′-DDE and reproductive end points are given in Table 3.

### Reproductive end points in relation to ER, AR, and AhR CALUX serum activity

In the subset of couples with valid TTP data and male CALUX activity data (*n* = 182), no consistent indications were found across regions of deviating fecundability according to ER, AR, or AhR CALUX assay activity. Moreover, sperm count, morphology, and motility were not related to agonistic, competitively enhanced, or antagonistic estrogenic serum activity across all regions or—with few exceptions—in any of the regions (Tables 4 and 5). Similar negative findings were observed for the AR and AhR CALUX measurements (data not shown). Summary findings with respect to other seminal characteristics are given in Tables 4 and 5 for the ER CALUX assays (data for other assays not shown). Comprehensive analyses of correlations between ER as well as AR and AhR xenobiotic activities and semen characteristics, including indicators of sperm DNA damage and abnormal sperm apoptosis, did not reveal consistent patterns of associations across regions. For details, the reader is referred to the original articles (Long et al. 2007; Toft et al. 2007).

## Discussion

Reports on secular trends in male reproductive health in the early 1990s became a major impetus for research on the hypothesis that male reproductive disorders may be related to xenobiotics with hormone-like properties. Two recent systematic reviews reexamined the evidence on the importance of xenohormone exposure in human reproductive health. Both reviews emphasize the current lack of explicit epidemiologic observations to validate the hypothesis (Storgaard et al. 2005; Vidaeff and Sever 2005). The purpose of the INUENDO project was to contribute additional insight into links between putative xenohormonal dietary exposure, as measured by POP markers in blood, and human fertility, as measured by an array of functional and biological indicators. To accomplish this objective approximately 5,000 women and spouses were enrolled in four cohorts in Greenland, northern, central, and eastern Europe. Below we discuss the coherence and synthesis of the main findings.

### Endocrine disruption

Experimental data have shown weak hormonal activity of the PCB congener CB-153 and the main DDT metabolite *p,p*′-DDE (Bonefeld-Jorgensen et al. 2001; Kelce et al. 1995; Moore et al. 1997), but our study does not provide strong or consistent evidence that these POP markers express hormone-like activity in humans at the prevailing exposure levels. Thus, the serum POP concentrations were not consistently related to either serum concetrations of sex hormones or to exogenous CALUX activities of serum across all study populations. However, an association between the concentration of *p,p*′-DDE in male serum and sex hormones in blood was observed in the Kharkiv region (Giwercman AH et al. 2006). Moreover, a weak increase in follicle-stimulating hormone with increasing CB-153 was observed in all regions but did not reach statistical significance in the pooled analysis. Whether these results have any bearing on impaired male reproductive function is not clear. One interpretation is that associations between POP and sex hormones are heterogeneous because of heterogeneous xenobiotic exposure that is only partly reflected by the POP markers measured in the present study. This is to some extent supported by the xenobiotic CALUX activities showing regional variation and weak correlations with POP markers. Another possibility is spurious associations emerging from a great number of explorative comparisons that have been carried out or uncontrollable bias inherent in study design and data collection. Because of the lack of strong and consistent associations, it is not possible to distinguish these different interpretations. But it should be kept in mind that cross-sectional associations based on point measurements of plasma concentrations of sex hormones reflect dynamic time-varying interactions and compensatory feedback mechanisms to a limited degree. Thus, no strong negative conclusions on the effect of POPs on hormonal homeostasis can be made from these findings.

### Post-testicular effetcs of POPs

In the present study, progressive sperm motility decreased with increasing POP blood levels in all four regions and seemed strongest among Inuits with rather high exposure to both POP markers. This finding is consistent with the results of six of seven cross-sectional studies (Aneck-Hahn et al. 2007; Dallinga et al. 2002; Danadevi et al. 2003; de Jager et al. 2006; Guo et al. 2000; Richthoff et al. 2003; Rozati et al. 2002). The two most recent studies carried out in Mexico and South Africa enrolled men with high environmental exposures and both demonstrated strong associations between serum concentrations of *p,p*′-DDE and various measures of sperm motility. Additional supportive evidence comes from two adult rat studies reporting reduced sperm motility after treatment with high doses of coplanar as well as noncoplanar PCBs (Hsu et al. 2003, 2004). Interestingly, the non-coplanar PCB congeners (CB-132 and CB-149) only affected sperm motility, whereas the coplanar dioxin-like PCB congener CB-77 also reduced sperm counts. The immotile testicular spermatozoa are gaining motility through slow passage through the epididymal tubules. Once ejaculated, secretions from the seminal vesicle and the prostate play a crucial role in energy supplies and composition of the seminal fluid and thereby in the ability of the spermatozoa to move (Elzanaty et al. 2006). Considering the consistent associations between CB-153 and sperm motility, it is of interest that NAG decreased with increasing CB-153 serum levels (but not *p,p*′-DDE serum levels), which is fairly consistent across the four regions (Elzanaty et al. 2006). NAG is excreted by the caudal part of the epididymis and is widely used as a marker of epididymal function in the clinical setting—low seminal levels, indicating impaired excretion and reduced epididymal function. Little is known about the physiologic role of the enzyme, but our study and an earlier study by Richthoff et al. (2002) show that NAG is weakly correlated with percentage motile sperm. For other seminal markers of epididymal and accessory sex gland function, findings were less consistent across regions.

Sex hormones regulate epididymal and accessory sex gland function. These organs express the estrogen, androgen, and the aryl hydrocarbon receptors. Some PCB congeners bind to the ER and AhR and elicit agonistic or antagonistic activity *in vitro.* Moreover, synthetic estrogens such as diethylstilbestrol and antiestrogens such as tamoxifen reduce epididymal and accessory sex gland weight when administered to adult rats in high doses (Elzanaty et al. 2006). However, as discussed above, this present study does not provide any explicit support for the assumption of endocrine disruption as the basic mechanism of action. Polymorphisms in the *AR* gene did not modify POP-related effects on sperm motility (Giwercman et al. 2008), and none of the CALUX activities that are assumed to represent the integrated xenohormone action from the mixture of pollutants in the serum extracts were consistently related to sperm motility across study populations. In addition, it can be argued that the estrogenic and androgenic equivalents contributed by xenobiotics in these highly exposed populations only are a few percent of the endogenous hormone activity, even when possible higher bioavailability of the xenobiotics is taken into consideration (Toft et al. 2007). However, in controlled rodent experiments, effects on reproductive function have been observed at very low exposure levels, particularly after exposure during the fetal and postnatal development periods (Anas et al. 2005; Mably et al. 1992)—an issue not explicitly addressed by this project. Thus, the picture with respect to mechanisms at the cellular level is far from clear, and the possibility cannot be excluded that POPs also exert toxic effects independent of hormonal actions. There is therefore a need for further research into the impact of POPs on epididymal function and gene expression.

### Gene–environment interaction

There are several earlier human studies on the effects of postnatal POP exposure on testicular function. In the study with subjects with the highest exposure to PCBs and polychlorinated dibenzo-*p*-dioxins after the Yucheng accident in Taiwan, abnormal sperm morphology and reduced sperm capacity to penetrate hamster eggs were found (Hsu et al. 2003). Two recent large cross-sectional studies of men with high environmental exposure to DDT in Mexico and South Africa failed, however, to demonstrate associations between serum concentrations of *p,p*′-DDE and sperm concentrations, whereas effects on semen volume and total sperm were found in one study (Aneck-Hahn et al. 2007) but not in another (de Jager et al. 2006). The lipid-adjusted serum concentrations were approximately 50–200 times higher in these groups of men than in men in our study. These findings seem consistent with the results in the present study where neither sperm count nor sperm morphology was related to POP exposure in any of the four study groups. However, among the subset of men with short *AR* CAG repeat length, which makes up about one-fifth of the entire study population, high levels of CB-153 were significantly related to low levels of sperm counts (Giwercman et al. 2007). This observation is an indication of gene–environment interaction between POPs and AR configurations. We acknowledge, however, that this finding emerges from a pooled analysis of four diverse study groups and calls for independent replication. The mechanism by which the CAG repeat length might modify the effect of POP on semen characteristics is not known. However, as the three-dimensional structure of the receptor is affected by the length of the CAG stretch, one could hypothesize that the strength of the POP binding, or of any necessary co-factor, is regulated by CAG number (Giwercman et al. 2007).

The INUENDO studies also indicate interactive effects on sperm chromatin integrity, although not necessarily gene–exposure interactions. The sperm chromatin structure assay measures two different types of sperm chromatin anomalies, one linked to DNA damage (%DFI) and the other reflecting abnormal condensation of proteins during the tight packaging of the sperm DNA (%HDS). Both types of chromatin anomalies are traced indirectly by *in situ* acid denaturation of sperm cells. Independent of sperm count, morphology, and motility, %DFI is related to male fertility, which starts decreasing when the percentage of abnormal sperm cells increases above approximately 20% (Spano et al. 2000). The %DFI measure is rather stable within subjects, and a number of studies of several European populations have shown remarkably homogenous distributions (Spano et al. 1998). Therefore, it is interesting that the %DFI levels were lower in Inuit men, indicating more “healthy” sperm chromatin. The sperm TUNEL assay demonstrated even more pronounced low levels of sperm DNA damage in Inuits. Comparable data have to our knowledge not been published previously. The low frequency of chromatin damage among Inuits, as demonstrated by two independent assays, could be genetic in origin, although dietary constituents as polyunsaturated fatty acids and selenium could also play a role. Interestingly, high serum concentrations of CB-153 were strongly related to low sperm integrity among European men, but there was no association among Inuit men. That exposure to POPs interferes with sperm chromatin integrity is further supported by the modifying effects of polymorphisms in the AR antigen (Giwercman et al. 2007). The finding that POP exposure increases the level of sperm DNA strand breaks is potentially worrying, as the risk for transmitting such chromosomal defects to the offspring are unknown. Animal studies have shown that epigenetic changes in the sperm DNA, evoked by *in utero* exposure to vinclozoline, may pass to and cause infertility in, at least, three subsequent generations (Anway et al. 2005). Further research is warranted to understand why Inuits have better sperm chromatin integrity than Europeans and why POPs are impairing chromatin integrity in Europeans but not among Inuits.

### Impact on fertility

Sperm count, morphology, motility, and chromatin integrity are all reliable and independent indicators of male fecundity (Bonde et al. 1998; Spano et al. 2000). The INUENDO studies demonstrated effects related to serum levels of POPs for three of these biological markers—most consistently for sperm motility. Nevertheless, the INUENDO study revealed no indications of delayed conception related to male or female POP serum levels except in Inuits where fecundability was reduced by 30% among the high-level-exposed groups. This finding was of borderline statistical significance and without strong exposure–response relationships but withstood a comprehensive analysis of potential bias and confounding factors including age and self-reported urogenital diseases. It is hard to explain why POPs interfere with fertility in only one of the four study groups. Neither exposure characteristcis nor POP-related effects on male biomarkers of fecundity offer any clear solutions to this enigma. To our knowledge, there are no comparable studies on fertility related to POP exposure among Inuit people, and the evidence from studies of other populations is conflicting (Toft et al. 2004). If not caused by bias inherent in the TTP methodology (Olsen et al. 1998) or residual confounding that escaped recognition, yet unknown gene–environement interactions should be identified in future research.

### Internal validity issues

The strengths of the INUENDO study include large exposure contrasts and large sample sizes relative to most end points, adherence to uniform design, protocol and data collection procedures, individual biological exposure markers, indicators of integrated xenohormone and dioxin-like actions, inclusion of a wide range of established and novel reproductive end points, analysis of selected *AR* polymorphisms, centralized data management and laboratory analyses with internal and external quality control, and an agreed upon protocol for the data analysis.

Several limitations must also be addressed. Few highly specific hypotheses were stated *a priori*, for example, whether PCBs or *p,p*′-DDE were to be considered the most important risk factor for the various reproductive end points. The same applies for the three CALUX assays, which represent a large range of possible risk-factor scenarios. It is important to acknowledge that most analyses were explorative. Multiple comparisons may constitute a serious risk for data-induced and *post hoc* interpretation of random associations (Smith 2001; Smith and Ebrahim 2002). Magnitude and statistical strength of associations, exposure–response relationships, gene polymorphisms, and biological plausibility has been considered throughout to help distinguish real from spurious associations. Another option to evaluate internal validity was to request consistent associations across the four study regions before assigning high credibility to the findings. Although still important, the obvious heterogeneity among regions with respect to POP and xenobiotic CALUX exposure profiles makes this “consistency-approach” less powerful than first anticipated. Systematic differences among regions in the genetic make-up that regulates metabolism of xenobiotics and reproductive functioning add further difficulties to the interpretation of heterogeneous associations. Even if in many aspects still elusive, environment–environment, gene–environment, and gene–gene interactions can play important roles in determining the relationship between exposure to environmental chemicals such as POPs and associated risks to human health and reproduction. Nevertheless, few disagree that associations that consistently survive background variation and heterogeneity, such as the relation between PCB and sperm motility, are more likely to be real. Furthermore, studies of associations stratified on relevant gene polymorphism may be important tools to advance knowledge in the field. Thus, the analyses of androgen gene polymorphisms in the INUENDO populations have for the first time demonstrated associations compatible with genetic modification of POP-related effects on male reproductive health.

Potential confounding factors were addressed systematically in all analyses according to a uniform protocol. Investigators considered well-established determinants as well as a number of hypothetical risk factors. Important determinants of the various outcomes may still escape adequate control because at present and to our best knowledge, they are unknown. Finally, the strong correlation between CB-153 and *p,p*′-DDE among Inuits and Swedish fishermen precludes allocation of effects to one or the other POP. Much weaker correlations yet large exposure ranges in the Warsaw and Kharkiv samples can, to some extent, resolve this problem.

### External validity issues

The sampling frame in three of the four regions was pregnant women and their partners. This should not be considered an important limitation to the external validity of all core studies. The couples enrolled consecutively also include those in the clinical setting who are labeled infertile, namely, those couples that took > 1 year to conceive. Approximately 15–20% of the “fertile” couples enrolled in this project would have been classified as infertile in the clinical terminology. Only a small minority of sterile couples are not represented in the database. This is not a problem unless the exposures being studied have an on–off effect, which is very unlikely (Baird 1988; Olsen 1999; Olsen et al. 1998). Among the few known human reproductive toxicants, tobacco smoking has been demonstrated with equal efficiency in pregnancy and population-based studies (Bolumar et al. 1996). Thus, the selection of pregnant women and their spouses for this study is not expected to reduce the chance of detecting the effects of POP on reproductive function.

There are several pieces of evidence that indicate reproductive organs are more sensitive to detrimental effects of chemicals during critical periods of fetal and postnatal development than during adult life (e.g,, Anas et al. 2005). It is therefore important to acknowledge that the research reviewed in this article does not explicitly address fetal exposures.

## Conclusion

The INUENDO study indicates that POPs may interfere with adult male reproductive function without major impact on fertility and sperm counts. However, subsets of men with specific polymorphisms in the *AR* gene seem more vulnerable. It is unknown if POP interference with sperm chromatin integrity and sperm DNA may have health consequences in the offspring. PCBs seem more to blame than DDT. No-effect thresholds could not be established. Findings provided limited direct evidence of xenobiotic disruption of the endocrine regulation. Further research is needed into POP-related effects on sperm chromatin integrity and epididymal function that include modifying effects related to *AR* polymorphisms.

## Correction

Figure 2C was incorrect in the manuscript originally published online and has now been replaced with the corrrect version.

## Figures and Tables

**Table 1:** The INUENDO study populations and measures of exposures and outcomes.

	Pregnant women and spouses attending antenatal care, 2002–2004					
Populations, exposure measures, and end points	Warsaw, Poland	Kharkiv, Ukraine	Greenland	Fishermen’s wives, Sweden	Fishermen, Sweden	All
Eligible target populations	690	2,478	665	1,439	2,783	8,055
Enrolled couples	472 (68%)	640 (26%)	598 (90%)	559 (35%)	—	2,269 (28%)
Blood samples, POPs (CB-153 and *p,p*′-DDE)						
Women	261	614	573	544	—	1,992
Men	257	287	440	—	189	1,172
Male hormones	144	215	325		190	874
CALUX						
Estrogen	99	89	74		100	362
Androgen	83	83	37		59	262
Dioxin-like	99	87	75		78	339
Eligible men addressed for the semen studies	690	640	256		2,783	4,369
Semen samples						
WHO	198	208	198		191	798
CASA	165	0	200		179	542
SCSA	143	208	200		184	736
TUNEL	134	134	195		166	634
APOPTOSIS	132	142	161		161	630
Y/X-SPERM	122	133	184		155	571
Accessory sex glands	187	203	184		158	732

Abbreviations: APOPTOSIS, percentage of ejaculated sperm cells expressing the Fas protein, as an indicator of apoptosis, and the Bcl-xL antigen, as an indicator of anti-apoptosis (Stronati et al. 2006); CALUX, chemical-activated luciferase gene expression; CASA, computer-aided sperm analysis; SCSA, sperm chromatin structure assay; TUNEL, terminal deoxynucleotidyl transferase dUTP nick end-labeling; WHO (World Health Organization), sperm count, morphology and motility measured according to the WHO 1999 guidelines (WHO 1999); Y/X SPERM, ratio between ejaculated spermatozoa with Y and X sex chromosome.

**Table 2:** Adjusted geometric mean values and linear regression coefficients of male reproductive hormones in serum, semen characteristics, and markers of epididymal and accessory sex gland function by categories of CB-153.

	Inuits			Europeans				
	CB-153 (ng/g lipid)			CB-153 (ng/g lipid)			Inuits	Europeans
	0–50	51–200	> 200	0–50	51–200	> 200	Linear regression coefficient (95% CI)	
	*n* = 10	*n* = 80	*n* = 104	*n* = 300	*n* = 182	*n* = 87	*n* = 194	*n* = 763
Male reproductive characteristics	(*n* = 19)	(*n* = 150)	(*n* = 145)	(*n* = 256)	(*n* = 183)	(*n* = 91)	(*n* = 314)	(*n* = 530)
Male reproductive hormones in serum^a^,^b^								
Follicle-stimulating hormone (IU/L)	4.2	4.1	4.5	3.9	4.3	4.8	0.04 (−0.02 to 0.1)	0.0008 (−0.06 to 0.07)
Luteinizing hormone (IU/L)	3.1	3.9^c^	4.1^c^	3.7	4.0	3.7	0.07^c^ (0.02 to 0.12)	−0.02 (−0.08 to 0.04)
Inhibin B (ng/L)	160	170	182	184	182	165	6.6 (−2.1 to 15.2)	−4.4 (−13 to 4)
Sex hormone−blinding globulin (mmol/L)	28	29	29	25	31^c^	32^c^	0.6 (−0.6 to 18)	2.1^c^ (0.7 to 35)
Free testosterone	1.63	1.73	1.75	1.87	1.68^c^	1.65^c^	0.01 (−0.01 to 0.04)	−0.05^c^ (−0.08 to −0.02)
Conventional semen characteristics								
Volume (mL)^d^,^e^	4.3	3.4	3.0^c^	3.1	3.6	3.3	−0.11^c^ (−0.2 to −0.04)	0.01 (−0.05 to 0.08)
Concentration (million/mL)^e^	58	52	53	46	53	64	0.03 (−0.1 to 0.2)	0.2 (0.0 to 0.2)
Count (million)^d^,^e^	229	274	149	142	185	200	−0.1 (−0.3 to 0.1)	0.1 (−0.0 to 0.2)
Normal sperm (%)	8.0	5.9	5.9	6.2	5.8	5.3	−0.0 (−0.1 to 0.1)	−0.0 (−0.1 to 0.1)
Progressive sperm (%)^e^,^f^	65	57	53^c^	60	57	51^c^	−4^c^ (−6 to −1)	−4^c^ (−6 to −1)
Sperm chromatin integrity^b^,^e^								
DNA fractionation index (%DFI)	8.0	7.6	7.5	9.9	12.8^c^	15.4^c^	−0.0 (−0.1 to 0.1)	0.2^c^ (0.1 to 0.2)
High DNA stainability (%HDS)	6.6	12.6^c^	11.0^c^	9.0	9.3	8.9	0.0 (−0.1 to 0.1)	−0.0 (−0.1 to 0.1)
DNA fractionation index [TUNEL (%)]	3.5	3.2	2.6	7.7	10.7^c^	12.0^c^	−0.1 (−0.3 to 0.0)	0.2^c^ (0.0 to 0.3)
Apoptotic markers^b^,^e^								
Fas positivity (%)	22.3	16.6	17.6	17.3	16.3	21.6	0.0 (−0.1 to 0.2)	−0.1 (−0.3 to 0.2)
Bcl-xL positivity (%)	12.7	12.6	10.3	16.6	16.6	20.6	−0.1 (−0.4 to 7.7)	0.4^c^ (0.1 to 0.7)
Epididymal and accessory sex gland function^b^,^d^,^e^								
Neutral α-glucosidase (mU/ejaculate)	25.0	16.6^c^	15.7^c^	18.5	26.8^c^	24.8^c^	−0.1^c^ (−0.2 to −0.0)	0.1 (−0.0 to 0.2)
Prostate-specific antigen (μg/ejaculate)	8.6	8.0^c^	8.0^c^	7.9	8.1^c^	7.9	−0.1 (−0.2 to 0.0)	0.0 (−0.1 to 0.1)
Zinc (μmmol/ejaculate)	6.6	4.8	4.2	4.5	5.9	5.1	−0.1 (−2.1 to 1.4)	0.0 (−0.1 to 0.1)
Fructose (μmmol/ejaculate)	71	43	44	38	44	35	−0.1 (−0.2 to 0.0)	−0.0 (−0.1 to 0.1)

Abbreviations: %DFI, percentage of sperm with denaturable DNA, mainly due to DNA damage (Spano et al. 2000); %HDS, percentage of sperm with high levels of green fluorescence, indicating immature sperm (Spano et al. 2000); *n,* number of semen samples; (*n* =), number of blood samples (analysis of reproductive hormones); TUNEL, terminal deoxynucleotidyl transferase dUTP nick end-labeling. All analyses among Europeans were adjusted for study group (Warsaw, Kharkiv, and Sweden).

a Adjustment by time of blood sampling (0800–1200 hours: yes/no).

b Adjustment by the logarithm of age (years).

c Mean values and linear regression coefficents associated with a *p*-value < 0.05 [exposed vs. reference group (CB-153, 0–50 ng/g lipid)].

d Samples with spillage were excluded.

e Adjustment by the logarithm of period of abstinence (day).

f Samples with a delay of > 1 hr from collection were excluded.

**Table 3:** Adjusted geometric mean values and linear regression coefficients of male reproductive hormones in serum, semen characteristics, and markers of epididymal and accessory sex gland function by categories of *p,p*′-DDE.

	Inuits			Europeans				
	*p,p*′-DDE (ng/g lipid)			*p,p*′-DDE (ng/g lipid)			Inuits	Europeans
	0–500	501–1,000	> 1,000	0–500	501–1,000	> 1,000	Linear regression coefficient (95% CI)	
	*n* = 82	*n* = 50	*n* = 62	*n* = 260	*n* = 188	*n* = 121	*n* = 194	*n* = 763
Outcome	(*n* = 155)	(*n* = 80)	(*n* = 78)	(*n* = 236)	(*n* = 177)	(*n* = 117)	(*n* = 314)	(*n* = 530)
Male reproductive hormones in serum^a^,^b^								
Follicle-stimulating hormone (IU/L)	4.5	4.3	4.2	3.8	4.3	4.5^c^	0.03 (−0.02 to 0.08)	0.06 (−0.01 to 0.14)
Luteinizing hormone (IU/L)	4.2	3.8	3.8	3.7	3.8	4.3^c^	0.05^c^ (−0.01 to 0.01)	0.05 (−0.01 to 0.11)
Inhibin B (ng/L)	181	177	170	189	177	166^c^	6.4 (1.7 to 13.8)	−13^c^ (−4 to −23)
Sex hormone–binding globulin (mmol/L)	28.2	29.0	29.1	26.3	28.3	30.8^c^	0.08 (−1.0 to 1.1)	1.3 (−0.3 to 2.8)
Free testosterone	1.79	1.72	1.70	0.59	0.57	0.55	0.02^c^ (0.0 to 0.04)	−0.02 (−0.05 to 0.01)
Conventional semen characteristics								
Volume (mL)^d^,^e^	2.8	3.2	3.5^c^	3.3	3.3	3.1	0.04 (−0.16 to −0.01)	−0.03 (−0.1 to 0.04)
Concentration (million/mL)^e^	55	51	52	49	53	58	−0.05 (−0.08 to 0.16)	0.14^c^ (0.02 to 0.27)
Count (million)^d^,^e^	150	150	180	165	165	160	−0.01 (−0.2 to 0.1)	0.12 (−0.03 to 0.27)
Normal sperm (%)	6.3	5.1	6.3	6.0	5.7	6.1	−3.2^c^ (−0.1 to 0.1)	0.03 (−0.09 to 0.15)
Progressive sperm (%)^e^,^f^	51	54	59^c^	59	57	55	−0.01 (−0.5.9 to -0.6)	−2.3 (−5.2 to 0.7)
Sperm chromatin integrity^b^,^e^								
DNA fractionation index (%DFI)	7.9	7.3	7.5	11.3	11.7	12.0	−0.01 (−0.1 to 0.1)	0.1 (−0.0 to 0.2)
High DNA stainability (%HDS)	10.2	12.6	11.5	9.3	8.7	9.3	0.01 (−0.1 to 0.1)	−0.02 (−0.1 to 0.1)
DNA fractionation index [TUNEL (%)]	3.9	4.3	4.3	9.3	9.2	9.5	−0.04 (−0.1 to 0.1)	0.0 (−0.1 to 0.2)
Apoptotic markers^b^,^e^								
Fas positivity (%)	18.3	19.0	17.7	16.9	17.4	19.9	−0.02 (−0.2 to 0.1)	0.2 (−0.1 to 0.4)
Bcl-xL positivity (%)	13.1	11.5	13.1	19.2	13.9	24.1	−0.03 (−0.3 to 0.1)	0.13 (−0.2 to 0.5)
Epididymal and accessory sex gland function^b^,^d^,^e^								
Neutral α-glucosidase (mU/ejaculate)	14.9	18.2	16.7	20.9	20.6	23.3	−0.05 (−0.1 to 0.1)	−0.0 (−0.1 to 0.1)
Prostate-specific antigen (μg/ejaculate)	2,930	3,270	3,210	2,940	2,550	3,000	−0.04 (−0.1 to 0.1)	−0.0 (−0.1 to 0.1)
Zinc (μmmol/ejaculate)	4.0	4.8	4.7	5.3	4.5	5.2	−0.01 (−0.2 to 0.1)	0.0 (−0.1 to 0.1)
Fructose (μmmol/ejaculate)	39	53	45	38	40	40	−0.05 (−0.2 to 0.1)	−0.0 (−0.2 to 0.1)

Abbreviations: %DFI, percentage of sperm with denaturable DNA, mainly due to DNA damage (Spano et al. 2000); %HDS, percentage of sperm with high levels of green fluorescence, indicating immature sperm (Spano et al. 2000); *n,* number of semen samples; (*n* =), number of blood samples (analysis of reproductive hormones); TUNEL, terminal deoxynucleotidyl transferase dUTP nick end-labeling. All analyses among Europeans were adjusted for study group (Warsaw, Kharkiv, and Sweden).

a Adjustment by time of blood sampling (0800–1200 hours: yes/no).

b Adjustment by the logarithm of age (years).

c Mean values and linear regression coefficents associated with a *p*-value < 0.05 [exposed versus reference group (CB-153, 0–50 ng/g lipid)].

d Samples with spillage were excluded.

e Adjustment by the logarithm of period of abstinence (day).

f Samples with a delay of > 1 hr from collection were excluded.

**Table 4:** Adjusted geometric mean values of semen characteristics according to serum agonistic estrogenic CALUX activity above and below average reference values.^a^

	Inuits		Europeans	
Semen characteristics	Increased activity [> 3.13 RLU (*n* = 16)]	Decreased activity [≤ 3.13 RLU (*n* = 56)]	Increased activity [> 3.13 RLU (*n* = 128)]	Decreased activity [≤ 3.13 RLU (*n* = 160)]
Conventional semen characteristics				
Volume (mL)^b^,^c^	3.9	2.9	3.0	3.4
Concentration (million/mL)^c^	41	55	51	53
Count (million)^b^,^c^	151	146	153	173
Normal cells (%)	6.8	5.3	6.3	5.0
Progressive motile (%)^c^,^d^	45	51	58	57
Seminal markers of accessory sex gland function^b^,^c^,^e^				
Neutral α-glucocidase (mU/ejaculate)	15	15	21	23
Prostate-specific antigen (μg/ejaculate)	2,161	3,364	3,260	2,896
Fructose (μmmol/ejaculate)	51	43	34	44
Zinc (μmmol/ejaculate)	2.7^f^	5.2	5.7	4.9
Sperm chromatin integrity^c^,^e^				
DNA fractionation index (%DFI)	4.3^f^	7.9	11.9	11.3
High DNA stainability (%HDS)	12.4	11.5	8.6	9.3
DNA fractionation index [TUNEL (%)]	1.8	2.5	10.9^f^	8.5
Apoptotic markers^c^,^e^				
Fas positivity (%)	11.6	12.9	21.2	16.0
Bcl-xL positivity (%)	7.7	16.8	17.3	20.5
Y chromosome sperm cells (%)	51.2	51.7	51.0	50.7

Abbreviations: %DFI, percentage of sperm with denaturable DNA, mainly due to DNA damage (Spano et al. 2000); %HDS, percentage of sperm with high levels of green fluorescence, indicating immature sperm (Spano et al. 2000); *n*, number of men; TUNEL, terminal deoxynucleotidyl transferase dUTP nick end-labeling.

a Reference 3.13 RLU, the mean value of solvent control samples.

b Samples with spillage excluded.

c Adjustment by the logarithm of period of abstinence (day).

d Samples with a delay of > 1 hr from collection excluded.

e Adjustment by the logarithm of age (years).

f Indicates *p* < 0.05 for increased versus decreased activity.

**Table 5:** Adjusted geometric mean values of semen characteristics according to serum competitive estrogenic CALUX activity above and below reference values.^a^

	Inuits		Europeans	
Semen characteristics	Increased activity [> 3.13 RLU (*n* = 6)]	Decreased activity [≤ 3.13 RLU (*n* = 66)]	Increased activity [> 3.13 RLU (*n* = 88)]	Decreased activity [≤ 3.13 RLU (*n* = 190)]
Conventional semen characteristics				
Volume (mL)^b^,^c^	3.8	3.1	3.1	3.3
Concentration (million/mL)^c^	57	56	52	52
Count (million)^b^,^c^	211	161	141	172
Normal cells (%)	6.7	6.3	5.4	5.5
Progressive motile (%)^c^,^d^	54	52	60	57
Seminal markers of accessory sex gland function^b^,^c^,^e^				
Neutral α-glucosidase (mU/ejaculate)	21	14	22	22
Prostate-specific antigen (μg/ejaculate)	3,833	2,970	3,489	2,887
Fructose (μmol/ejaculate)	56	45	41	40
Zinc (μmmol/ejaculate)	6.3	4.4	5.8	5.0
Sperm chromatin integrity^c^,^e^				
DNA fractionation index (%DFI)	4.9	7.1	11.3	11.6
High DNA stainability (%HDS)	10.8	11.1	8.8	9.3
DNA fractionation index [TUNEL (%)]	1.4	2.5	8.3	9.5
Apoptotic markers^c^,^e^				
Fas positivity (%)	11.4	13.0	14.2	19.0
Bcl-xL positivity (%)	19.8	14.4	14.8	20.0
Y chromosome sperm cells (%)	53.6	51.2	50.9	50.7

Abbreviations: %DFI, percentage of sperm with denaturable DNA, mainly due to DNA damage (Spano et al. 2000); %HDS, percentage of sperm with high levels of green fluorescence, indicating immature sperm (Spano et al. 2000); *n*, number of men; TUNEL, terminal deoxynucleotidyl transferase dUTP nick end-labeling.

a Reference 3.13 RLU, the mean value of solvent control samples.

b Samples with spillage excluded.

c Adjustment by the logarithm of period of abstinence (day).

d Samples with a delay of > 1 hr from collection excluded.

e Adjustment by the logarithm of age (years).
